# Recuperative Hostels

**Published:** 1917-05-19

**Authors:** 


### RECUPERATIVE HOSTELS.

### The Hankering after Euphemisms.

A project is on foot, initiated by Sir Frederick Milner, who has shown again and again his interest in men disabled in the war, and by Sir Arthur Sloggett, who is in a position to know the facts, to institute what are to be called Recuperative Hostels for the treatment of men who are described as "nerve-shattered" or "nerve-wrecked" by experiences in the war. Sir Frederick Milner, who is the chairman of the committee that is appealing for funds for this purpose, tells us that men in the condition described "have been, and are being, invalided out of the Services in large numbers, with no prospect before them but the workhouse or asylum, or a miserable existence as a burden to themselves and their relations." A letter in the *Pall Mall Gazette* asks why such unfair discrimination exists between the kind of treatment meted out to wounded and unwounded men when equally affected in the matter of nerve-strain. "The unwounded have fought as heroically as the wounded; it is the mere chance of fate. Yet how different, how ominous in all respects is the prospect with which they find themselves confronted! . . . The physically disabled are the recipients of the nation's bounty; those disabled through transient nerve exhaustion are doomed to a position which associates them unwarrantably with the stigma of lunacy. Their reward is not honour, but disgrace."

The view taken in this letter is deplorable, and whatever disgrace there is attaches not to the insane, but to the person who considers insanity disgraceful. One poison circulating in a man's blood affects most his great toe, and then that person suffers from podagra, a manifestation of gout, that is considered rather honourable than disgraceful. Another poison circulating in his blood produces septicæmia, or furunculosis; but these are not considered disgraceful. Another poison circulating in the blood affects the peripheral nervous system, and produces dropped wrist or colic; and these are not considered disgraceful. Yet another poison affects a portion of the central nervous system, and produces tetanus, and this is not considered disgraceful. Another poison affects another portion of the central nervous system, and produces insanity, and this, forsooth, is considered disgraceful! How grossly ignorant, how shockingly prejudiced, must he not be who so considers it! If the poison happens to be that of smallpox or of typhoid fever, or influenza, and if it affects the brain so as to produce insanity, there is nothing disgraceful in the insanity as long as the fever lasts; it is then "only" delirium, and is quite respectable; but if it continues after the fever subsides, then it ceases to be "only" delirium, and becomes madness; and then it ceases to be respectable, and becomes disgraceful! What muddy thought is here! What muddleheadedness! The writer in the *Pall Mall Gazette* entreats us to bring common sense to bear upon the problem. Common sense! What title has the writer of such stuff as this to advocate the employment of common sense? Let him look at home and cultivate some for himself before he preaches it to other people.

But passing this, is it a fact that the unwounded but nerve-shattered man is cast off by the War Office, and has nothing done for him? This is implied both in the circular of Sir Frederick Milner and in the *Pall Mall Gazette* letter. The name of Sir Arthur Sloggett would seem to vouch for the statement; but in the first place Sir Arthur Sloggett is fully employed at the Front, and is scarcely in as good a position to know what happens to men discharged from the Army after their return home, or to know in what condition they are discharged, as the head of the Medical Service in this country; and Sir Alfred Keogh's name does not appear on the committee or among the list of patrons. Moreover, though Sir Arthur Sloggett is a patron of the movement, and no doubt sympathises with the object of providing hostels, he does not endorse the statement, if it is a statement, that shell-shocked sailors and soldiers are treated with less sympathy by the War Office than the wounded. The Hospital is not disposed to accept this as true until it has been definitely asserted by a responsible person and proof is forthcoming. The Committee of Reference appointed jointly by the Royal Colleges of Physicians and Surgeons made some time ago, we believe, a formal recommendation that men should not be invalided out of the Army until the resources of treatment had been exhausted, and the ground on which the recommendation was based was that unless kept under military discipline, the men would shirk or neglect the treatment recommended, which is often tedious and often irksome. Whether this recommendation has been adopted and acted upon we do not know; but if it has, it is clear that a man will be entitled to all the benefits of the best treatment to be obtained until patient trial has shown that improvement is not to be expected.

If improvement is not to be expected, the man cannot be kept on the muster roll indefinitely. A time must come when he must be discharged, and it is very right and proper that there should be a hostel or other institution to which he can go, if it is suitable for him to go there; but what is the sense of objecting to a man being sent to a lunatic asylum if he is in fact insane? If he is mad, the fact that his madness was caused by shell shock or other experience of war does not render him any the less mad, nor does it render those whose madness is due to other causes unfit companions for him. There is nothing disgraceful, except in the eyes of very silly people, in being mad; and there is nothing more disgraceful in being mad from an unknown cause, or from fever, or influenza, than in being mad from shell shock. In the present state of the Lunacy Law, men who are discharged mad from the Navy or Army cannot be received in numbers in a hostel without breach of the law; and if the law is to be altered so that they can be received therein, then those residents who are not mad will be subjected to the "disgrace" of associating with those who are, and, moreover, will have been received into an institution specially adapted for the reception of the insane. These "hostels," or "convalescent homes," are to be "without suspicion of the taint of lunacy." Very well, but how is the taint of lunacy to be avoided if lunatics discharged from the Navy and Army are admitted into them? To speak of the "taint of lunacy" is an expression of which any man ought to be ashamed, and any enlightened man, any man of common sense, is ashamed—when he finds other people using it. No such man would ever use it himself.

An absurd notion is prevalent that asylums are mere receptacles for the incurably mad to be kept in till they die. In fact, about 50 per cent. of the patients admitted into lunatic asylums recover, are discharged, and return to their normal life in the world. It is commonly supposed that no one is placed in an asylum unless he is not only incurable and irrecoverable, but dangerous to himself or others. This supposition is prevalent not only among the laity, but among medical men; and not only among medical men, but among some, at least, who pose as experts in insanity: and it is not at all infrequent among lawyers, and has been asserted in Courts of Law. It is utterly opposed to the truth. The words of the magistrate's order, under which alone a patient may be placed in an asylum, are that he is a proper person to be detained under *care and treatment*. He is placed in an asylum to be taken care of and treated, and under this care and treatment some 50 per cent. recover. For what purpose are patients to be sent to the "Recuperative Hostels"? Surely for care and treatment. Then in what respect are these hostels to be different from asylums? In name only. The same law must be obeyed, the same formalities must be observed, the same construction, rules, and arrangements must be made. They will be asylums in everything but name, and the name of "hostel" will soon equally repel. But there is no reason why the name of "asylum" should any longer repel. Many asylums now call themselves by the absurd title of "mental hospital," in the vain endeavour to free themselves from the "stigma" and the "taint" and the "disgrace," which will very soon cling about "mental hospital" and "hostel" as it clings about "asylum," which was substituted for "madhouse" for the very same purpose. The proper way to go to work is to disabuse the minds of silly people that there is any "stigma," or any "taint," or any "disgrace" attaching to madness, or to the places in which madmen reside and are cared for and treated.

